# Clinical Spectrum in a Cohort of Patients With High Fecal Calprotectin Levels

**DOI:** 10.7759/cureus.11314

**Published:** 2020-11-03

**Authors:** Lena Jafri, Ayra Siddiqui, Sabeeh Sidddique, Om Parkash, Rizwana Kausar, Hafsa Majid

**Affiliations:** 1 Section of Chemical Pathology, Department of Pathology and Laboratory Medicine, Aga Khan University Hospital, Karachi, PAK; 2 Department of Pathology and Laboratory Medicine, Karachi Grammar School, Karachi, PAK; 3 Section of Histopathology, Department of Pathology and Laboratory Medicine, Aga Khan University Hospital, Karachi, PAK; 4 Department of Gastroenterology, Aga Khan University Hospital, Karachi, PAK

**Keywords:** calprotectin, abdominal cramps, diarrhea, weight loss, inflammatory bowel diseases

## Abstract

Introduction: Distinguishing between inflammatory bowel disease (IBD) and functional gastrointestinal disorders is a diagnostic challenge. The need for non-invasive biomarker as a diagnostic tool in the assessment of gastrointestinal symptoms is required. The objectives of current study were to determine the spectrum of clinical features in patients tested for fecal calprotectin presenting with high levels and to compare calprotectin levels among already diagnosed patients known to have IBD as per biopsy findings and documented on patients' file with newly presenting patients who were being investigated and did not have a diagnosis.

Methods: This retrospective cross-sectional study was conducted in the Department of Pathology and Laboratory Medicine and Department of Medicine, Aga Khan University, Karachi, Pakistan from January 2017 to December 2019. Subjects tested for fecal calprotectin who had elevated fecal calprotectin levels (n = 150) were included in the current study. Each patient deposited a random stool sample in an airtight container for calprotectin analysis. Biochemical analysis of calprotectin was performed by enzyme-linked immunosorbent assay using epitope calprotectin test kit (Epitope Diagnostics, Italy) on ETI-Max 3000 immunoassay analyzer (DiaSorin, Italy). A structured history form was used for data collection.

Results: One hundred and fifty patients were available for inclusion in the ﬁnal analysis. Majority of the patients (n = 117, 78%) were adults (>18 years of age), and 52.7% (n = 79) were females. Median fecal calprotectin (IQR) was 317.3 μg/g (549.10 - 239.2 μg/g) in children (n = 33) and 305 μg/g (609.9 - 201.6 μg/g) in adults; the difference was statistically non-significant (p value > 0.05). On categorization according to disease, fecal calprotectin levels were significantly elevated (p value = 0.033) in IBD patients compared to normal subjects, 644 μg/g (644 - 587.8 μg/g) vs 308.5 μg/g (505.4 - 233.8 μg/g), respectively. Diarrhea (n = 13, 38.4%), abdominal cramps (n = 12, 36.4%), and weight loss (n = 11, 33.3%) were the most common complaints noted in children with high fecal calprotectin levels, whereas in adults, abdominal cramps (n = 60, 51.3%), diarrhea (n = 59, 50.4%), and weight loss (n = 46, 39.3%) were the common complaints. The median fecal calprotectin levels in children already known to have IBD (n = 3) were higher than the levels noted in children with no diagnosis (n = 30); p value > 0.05. Similarly, median fecal calprotectin levels in adults with IBD (n = 28) were higher than the levels noted in patients with no specific diagnosis (n = 91), 400.7 μg/g (656.6 - 244.3 μg/g) vs 302.7 μg/g (564.6 - 206 μg/g); p value > 0.05.

Conclusion: Current study affirms that the fecal calprotectin test can be used in identifying IBD patients in all age groups.

## Introduction

Inflammatory bowel disease (IBD) is a chronic disorder that includes two main forms of chronic gastrointestinal (GI) inflammation: ulcerative colitis (UC) and Crohn's disease (CD). Internationally, the incidence of IBD is approximately 0.5-24.5 cases per 100,000 person-years for UC and 0.1-16 cases per 100,000 person-years for CD [1]. The overall annual prevalence of IBD in the United States (US) is nearly 396 cases/100,000 persons. Its estimated incidence is 1.3% of US adults (3 million), reported by the Centers for Disease Control and Prevention (CDC) in 2015 [2]. Not enough data is available in this regard in local literature, and the prevalence is unknown. Differentiating between IBD and functional gastrointestinal disorders (bowel disorders with impaired movement of the intestines when no structural abnormalities are found) is usually a diagnostic caveat. Abdominal pain, bloating, and diarrhea are common nonspecific GI symptoms in both pediatric and adult population. Invasive procedures like endoscopy, colonoscopy, and histology are sometimes required to define the underlying etiology, which is generally challenging. Due to the nonspecific GI symptoms of CD and UC, several other differentials such as irritable bowel syndrome (IBS) must be considered before establishing a confirmed diagnosis of IBD, particularly in the absence of typical endoscopic findings and in populations at higher risk for other diagnoses. The introduction of biomarkers as non-invasive diagnostic tools in the assessment of various GI symptoms may ultimately decrease the use of invasive, complicated, and costly procedures like endoscopy and biopsy.

Several laboratory studies, like pro-inflammatory markers, are of value in assisting with the management of IBD and providing supporting information. However, no single laboratory investigation can confirm the diagnosis of IBD. One may use the laboratory values as surrogate markers for inflammation and nutritional status and look for vitamins and minerals deficiencies. The biomarkers, such as erythrocyte sedimentation rate (ESR) and C-reactive protein (CRP), have been investigated to help diagnose IBD. Still, the findings were not promising [3,4]. Care must be taken to prevent unnecessary testing as well as delayed or missed diagnosis. In health care, both the physicians and the patients would benefit from non-invasive and specific screening tests. Fecal calprotectin appears to be one promising surrogate biomarker that may assist in the diagnostic workup of patients with vague GI symptoms to select them for further workup and invasive testing [5]. Fecal calprotectin has also been proposed as a candidate screening marker for discrimination between IBD and IBS.

Calprotectin is a marker of inflammation from the S100 calcium-binding protein family. It is expressed mainly by granulocytes and, to a lesser extent, by epithelial cells, reactive macrophages, and monocytes [6]. In neutrophils, approximately 60% of the total cytoplasmic protein content is made up of calprotectin [7]. The fecal calprotectin concentration is directly proportional to the neutrophils present in the gastrointestinal mucosa and is used as an indirect marker of intestinal inflammation [8]. However, the predictive value of fecal calprotectin to distinguish functional from organic GI disorders needs further evaluation. Several studies have found that the measurement of fecal calprotectin is useful for the early diagnosis of IBD [9-10]. The literature on the clinical utility of fecal calprotectin from this part of the world is scarce. The aim of the current study is to determine the spectrum of clinical features in patients with high fecal calprotectin and to compare calprotectin levels between already diagnosed patients known to have IBD as per biopsy findings and documented on patients' clinical notes with newly presenting patients who were being investigated and did not have a diagnosis.

## Materials and methods

A cross-sectional observational study was conducted at the Section of Chemical Pathology, Department of Pathology and Laboratory Medicine in collaboration with the Department of Medicine from January 2017 to December 2019. Before the initiation of the study, our investigation protocol was reviewed and approved by the Ethics Committee at Aga Khan University (ERC number: 2019-1616-5031), and the study was conducted per the Helsinki Declaration. All included patients and parents or guardians of children recruited in the study provided informed consent over the telephone at enrollment after being informed of the study purpose. Inclusion criteria were all subjects tested for fecal calprotectin who had elevated fecal calprotectin levels. This study included both adults and children (less than 18 years of age). Consecutive purposive sampling technique was used, and subjects fitting into the inclusion criteria during the study period were included. Subjects with incomplete data or repeat testing were excluded. A structured clinical history form was used for data collection via telephone history and review of clinical data on laboratory information management system, which included clinical history, biochemical data (fecal detailed report, occult blood), and findings of intestinal biopsy if performed through the telephonic interview. Patients were categorized into two groups as: (1) already diagnosed patients known to have IBD based on biopsy findings and documented on their clinical charts and (2) newly presenting patients who were being investigated and did not have a diagnosis. 

Analysis of fecal calprotectin consists of an extraction step on a random stool sample followed by quantification by immunoassay. Biochemical analysis of calprotectin was performed by enzyme-linked immunosorbent assay using epitope calprotectin test kit (Epitope Diagnostics, Italy) on ETI-Max 3000 immunoassay analyzer (DiaSorin, Italy). To validate the fecal calprotectin results, high- and low-quality control material was run with every batch. Fecal calprotectin cutoff of 43.2 μg/g was used as per product insert from the calprotectin test kit to distinguish between IBS (nonorganic disease) and IBD (organic disease) for both adults and pediatric subjects. Additionally, samples were also received for external quality assessment from the College of American Pathologists during the study period, and all results were acceptable.

Data were compiled into Microsoft Excel for analysis, and statistical analysis was performed with Statistical Package for the Social Sciences (SPSS, IBM Corp., NY) version 22. Mean and standard deviation for parametric and median with interquartile range for non-parametric quantitative variables were generated, while frequency and percentage of qualitative variables were generated. For comparison of numerical and categorical variables, the Mann-Whitney U test and the chi-squared test or Fischer's exact test were applied as appropriate. Independent “t” test was performed to compare the calprotectin levels between diagnosed patients with IBD as per biopsy findings, and newly presenting patients were being investigated who did not have a diagnosis. In each analysis, p values less than 0.05 were considered statistically significant.

## Results

**Figure 1:**
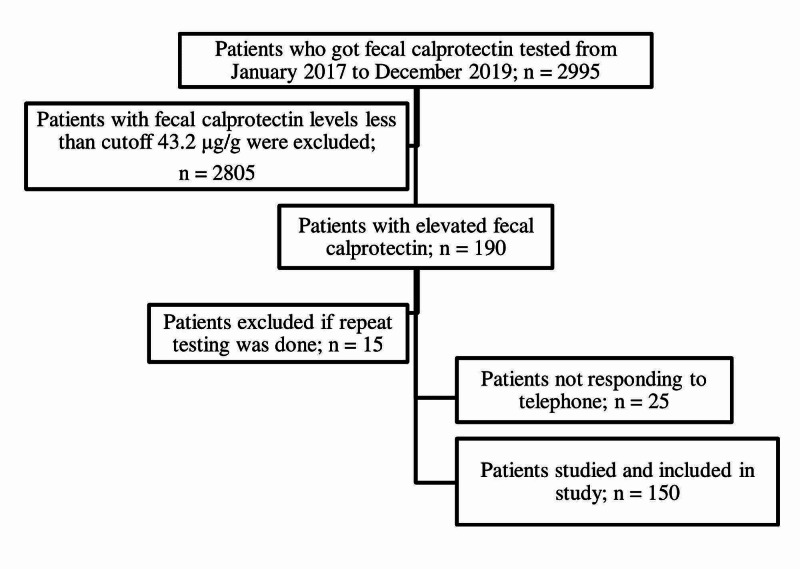
Flowchart detailing patients’ selection in the study.

A total of 2995 fecal calprotectin tests were performed in the clinical laboratory of Aga Khan University in three years from January 2017 to December 2019. Among the 190 patients initially included who had high fecal calprotectin levels, 40 were excluded as those were repeat testing samples or patients who did not respond to telephonic contact (three times). Therefore, 150 patients were available for inclusion in the ﬁnal assessments of the study outcomes, as shown in Figure 1.

The baseline demographic characteristics of the 150 patients included in the study are shown in Table 1. Out of the total 150 patients recruited, the majority (n = 117, 78%) were adults (>18 years of age). The mean age was 32.1 ± 18 years, and 52.7% (n = 79) patients were females. Abdominal cramps (n = 72, 48%), diarrhea (n = 71, 48%), weight loss (n = 57, 38%), blood in stool (n = 47, 31.3%), and bloating (n = 45, 30%) were the most common signs and symptoms noted in the overall study population studied. In most patients (n = 104, 69.3%), medical treatment was not administered. However, the majority of those on treatment were receiving antidiarrheal with antibiotics. Among all 150 patients, fecal calprotectin levels ranged from 46.3 to 6284 μg/g. Fecal calprotectin levels in children were higher as compared to adults, but the difference was statistically non-significant (p value > 0.05). On categorization according to disease, fecal calprotectin levels were significantly elevated (p value = 0.033) in IBD patients compared to subjects with no established diagnosis, 644 μg/g (644 - 587.8 μg/g) vs 308.5 μg/g (505.4 - 233.8 μg/g), respectively.

**Table 1:** Demographics as well as clinical and biochemical characteristics of study subjects.

Variables	Study Subjects			p values
	Overall n = 150	Pediatric n = 33	Adults n = 117	
Mean age in years (SD)	32.1 (18)	9.7 (4.9)	38.4 (15.1)	0.00
Female; n (%)	79 (52.6)	20 (60.6)	59 (50.4)	0.30
Abdominal cramps; n (%)	72 (48)	12 (36.4)	60 (51.3)	0.38
Weight loss; n (%)	57 (38)	11 (33.3)	46 (39.3)	0.92
Blood in stool; n (%)	48 (32)	9 (27.7)	39 (33.3)	0.45
Bloating; n (%)	45 (30)	9 (27.7)	36 (54.5)	0.64
Bleeding ulcers; n (%)	44 (29.3)	8 (24.2)	36 (30.8)	0.25
Diarrhea; n (%)	72 (48)	13 (38.4)	59 (50.4)	0.45
Fatigue; n (%)	38 (25.3)	8 (24.2)	30 (25.6)	0.89
Decreased appetite; n (%)	30 (20)	8 (24.2)	22 (18.8)	0.23
Mucus in stool; n (%)	25 (16.6)	7 (21.2)	18 (15.4)	0.61
Alternating constipation and diarrhea; n (%)	25 (16.6)	6 (18.2)	19 (16.2)	0.92
Constipation; n (%)	15 (10)	2 (6.1)	13 (11.1)	0.80
Tenesmus; n (%)	18 (12)	2 (6.1)	16 (13.7)	0.47
Fever; n (%)	18(12)	5 (15.2)	13 (11.1)	0.80
Dyspepsia; n (%)	18 (12)	3 (9.1)	15 (12.8)	0.40
Black stool; n (%)	14 (9.3)	2 (6.1)	12 (10.3)	0.62
Belching; n (%)	11 (7.3)	2 (6.1)	9 (7.7)	0.91
Diagnosed inflammatory bowel disease; n (%)	19 (12.6)	2 (6.1)	17 (14.5)	0.27
Diagnosed ulcerative colitis; n (%)	10 (6.6)	1 (3.0)	9 (7.7)	
Median fecal calprotectin (Q3-Q1) in μg/g	306.7 (588.2 - 212.5)	317.3 (549.10 - 239.2)	305 (609.9 - 201.6)	0.55

Pediatric subjects

Mean age (SD) of the 33 children included was 9.7 (±5) years, as described in Table 1. Median fecal calprotectin was 317.3 μg/g (549.10 - 239.2 μg/g) in children. Diarrhea (n = 13, 38.4%), abdominal cramps (n = 12, 36.4%), and weight loss (n = 11, 33.3%) were the most common complaints found in children with elevated fecal calprotectin levels. In most children (n = 22, 66.6%), medical treatment was not initiated at the time of recruitment. As shown in Table 2, the median fecal calprotectin levels in children already known to have IBD (n = 3) were higher than the levels noted in children with no diagnosis (n = 30), but the difference was statistically non-significant (p value > 0.05).

**Table 2:** Describing clinical and biochemical features of patients already known to have inflammatory bowel disease (IBD) in comparison to newly presenting patients with no confirmed diagnosis.

	Pediatric, n = 33		p values	Adults, n = 117		p values
Variables	Patients already known to have IBD, n = 3	Newly presenting patients with no diagnosis, n = 30		Patients already known to have IBD, n = 26	Newly presenting patients with no diagnosis, n = 91	
Mean age (SD)	9 (7)	9.8 (4.8)	0.78	38 (14.6)	38.5 (15.4)	0.88
Female; n (%)	1 (33.3)	19 (63.3)	0.54	12 (46.2)	47 (51.6)	0.66
Medications:						
Not on any treatment; n (%)	1 (33.3)	21 (70)	0.006	12 (46.2)	70 (76.9)	0.00
Antidiarrheal + Antibiotics; n (%)	0	3 (10)		1 (3.8)	10 (11)	
Antidiarrheal; n (%)	0	1 (3.3)		1 (3.8)	3 (3.3)	
Antidiarrheal + Ispaghula powder; n (%)	0	0		0	1 (1.1)	
Antibiotic; n (%)	0	3 (10)		1 (3.8)	2 (2.2)	
5-Aminosalicylate (5-ASA); n (%)	0	0		6 (23.1)	1 (1.1)	
Corticosteroids; n (%)	0	0		1 (3.8)	0	
5-ASA + Corticosteroids; n (%)	0	0		3 (11.5)	0	
Antibiotics + Herbal; n (%)	0	0		0	1 (1.1)	
Herbal; n (%)	0	0		1 (3.8)	2 (2.2)	
AntiTB; n (%)	0	2 (6.7)		0	0	
Antidepressants; n (%)	0	0		0	1 (1.1)	
Antidiarrheal + Antiallergy	1 (33.3)	0		0	0	
Median fecal calprotectin (Q3-Q1) in μg/g	644 (644 - 587.8)	308.5 (505.4 - 233.8)	0.06	369 (692.4 - 223.7)	296 (587.6 - 197.9)	0.16

Adult subjects

Mean age (SD) of adults (n = 117) was 36.2 (14.7) years. Median fecal calprotectin was 305 μg/g (609.9 - 201.6 μg/g) in this adult group. Abdominal cramps (n = 60, 51.3%), diarrhea (n = 59, 50.4%), and weight loss (n = 46, 39.3%) were the most common complaints found in adult patients with elevated fecal calprotectin levels. Majority of the adult patients (70.8%) were not on any medical treatment at the time of recruitment. The median fecal calprotectin levels in adult patients already known to have IBD (n = 29) were higher than the levels noted in patients with no specific diagnosis (n = 91), 369 μg/g (692.4 - 223.7μg/g) vs 296 μg/g (587.6 - 197.9 μg/g), but the difference was statistically non-significant (p value = 0.16).

## Discussion

In this study, we observed that median fecal calprotectin levels in diagnosed IBD patients were high in comparison to the group with no definitive diagnosis. In children, the fecal calprotectin levels were significantly higher compared to patients with no established diagnosis, whereas in adults, fecal calprotectin levels although higher in IBD group were not statistically significant. Analogous findings were reported by Carroccio et al. who observed higher diagnostic accuracy of fecal calprotectin in children as compared to adults. The sensitivity and specificity of fecal calprotectin in children were 100% and 95%, respectively, as reported by them, whereas in adults it was 64% and 80%, respectively. The reasons for a greater number of false-positive results in adults was the use of certain drugs such as aspirin, nonsteroidal anti-inflammatory drugs of concomitant occurrence of other inflammatory, or autoimmune diseases [11]. Ashorn et al. studied the clinical utility of fecal calprotectin and reported that children and adolescent IBD patients had higher fecal calprotectin levels (≥100 μg/g) compared to patients with no established diagnosis presenting with colitis [12].

Similarly, another study done in children by Henderson et al. reported that fecal calprotectin levels were significantly raised in IBD patients compared to non-IBD controls [13]. However, they found no statistically significant difference in fecal calprotectin levels in CD or UC patients. They also stated that fecal calprotectin was better than CRP and white cell count in predicting IBD-suspected patients eligible for further endoscopic and histologic investigation. A meta-analysis by Henderson et al. reported that pooled sensitivity of fecal calprotectin for diagnosing IBD was 0.978 (95% confidence interval [CI], 0.947-0.996) and pooled specificity was 0.682 (95% CI, 0.502-0.863) with a positive likelihood ratio of 3.07 [14]. Mooiweer et al. reported that fecal calprotectin levels remain low in IBD patients with remission and can be used to follow patients for clinical improvement [15]. These findings show that fecal calprotectin is a better marker than the most commonly available pro-inflammatory markers, e.g., CRP, ESR in identification, and prognosis of IBD patients.

However, till to date the gold standard for diagnosing IBD and differentiating UC and CD is ileocolonoscopy with histological studies. In the large majority of patients suffering from IBD, it is possible to reliably differentiate between UC and CD based on the histological features. In UC, the changes are limited to the mucosa. There is ulceration, crypt distortion and disarray, cryptitis, and crypt abscesses [16]. Prominent basal plasmacytosis is also present. Paneth cell metaplasia, especially in the left colon, is another feature that may be seen in the cases of UC. CD, on the other hand, leads to transmural inflammation of the bowel wall. In addition to the mucosal ulceration and architectural distortion, non-caseating granulomata are an important histological feature of Crohn's [17]. Fissures may also be seen extending from the mucosa into the submucosa. Submucosa and muscularis propria may show fibrosis and scattered discrete lymphoid aggregates.

In some cases, the biopsy may show overlapping features between UC and CD and the histological distinction between the two may not be possible, therefore being labeled as indeterminate colitis [18-20]. It is understood that the findings of one diagnostic test cannot replace another test. From a practical point of view, fecal calprotectin measurement is quicker, non-invasive, and more patient-friendly than the standard endoscopic procedures. In the past decade, several researchers have investigated the role of fecal calprotectin in distinguishing IBD from IBS, and these have been summarized in several recent meta-analyses by Gisbert and McNichol. Combined data from 2475 patients obtained a mean sensitivity of 83% and specificity of 84% for calprotectin to distinguish organic and nonorganic disease [21]. Based on the findings from the current study, it is not possible to suggest recommendations to use fecal calprotectin as a screening test for distinguishing IBD from non-IBD cases because of several limitations of the study. There were several limitations of the study, including small sample size, cross-sectional study design, and non-availability of histological findings in all cases. Larger prospective studies are needed to evaluate further the role of fecal calprotectin in screening and prognosis of IBD patients.

## Conclusions

In conclusion, the present study affirms that the fecal calprotectin levels were higher in patients with IBD compared to those with no established diagnosis. The performance of this test is better in children with IBD compared to adults. Moreover, since not all IBD patients had significantly high fecal calprotectin levels, endoscopy and histology remain the gold standard for labeling any patients with IBD, and the fecal calprotectin test can be utilized in predicting patients suspected with IBD that can be referred for further invasive investigations.
